# Oxalate of Cerium in Pertussis

**Published:** 1879-10

**Authors:** 


### OXALATE OF CERIUM IN PERTUSSIS.

Dr. Morjé of New York, in the *Medical Record*, claims for this remedy:

1. That it decreases the attacks and thereby reduces the volume of the disease, and often checks it instantly.
2. That it is easily administered, only one dose being required in twenty-four hours.
3. That it secures nocturnal quietude.
4. That the possibility of complications is lessened.

It is given in doses of from one to three grains, before eating in the morning.

